# Bortezomib Resistance Can Be Reversed by Induced Expression of Plasma Cell Maturation Markers in a Mouse *In Vitro* Model of Multiple Myeloma

**DOI:** 10.1371/journal.pone.0077608

**Published:** 2013-10-29

**Authors:** Holly A. F. Stessman, Aatif Mansoor, Fenghuang Zhan, Michael A. Linden, Brian Van Ness, Linda B. Baughn

**Affiliations:** 1 Department of Genetics, Cell Biology and Development, University of Minnesota, Minneapolis, Minnesota, United States of America; 2 Department of Internal Medicine, University of Iowa Carver College of Medicine, Iowa City, Iowa, United States of America; 3 Department of Laboratory Medicine and Pathology, University of Minnesota, Minneapolis, Minnesota, United States of America; Children’s Hospital Boston, United States of America

## Abstract

Multiple myeloma (MM), the second most common hematopoietic malignancy, remains an incurable plasma cell (PC) neoplasm. While the proteasome inhibitor, bortezomib (Bz) has increased patient survival, resistance represents a major treatment obstacle as most patients ultimately relapse becoming refractory to additional Bz therapy. Current tests fail to detect emerging resistance; by the time patients acquire resistance, their prognosis is often poor. To establish immunophenotypic signatures that predict Bz sensitivity, we utilized Bz-sensitive and -resistant cell lines derived from tumors of the Bcl-X_L_/Myc mouse model of PC malignancy. We identified significantly reduced expression of two markers (CD93, CD69) in “acquired” (Bz-selected) resistant cells. Using this phenotypic signature, we isolated a subpopulation of cells from a drug-naïve, Bz-sensitive culture that displayed “innate” resistance to Bz. Although these genes were identified as biomarkers, they may indicate a mechanism for Bz-resistance through the loss of PC maturation which may be induced and/or selected by Bz. Significantly, induction of PC maturation in both “acquired” and “innate” resistant cells restored Bz sensitivity suggesting a novel therapeutic approach for reversing Bz resistance in refractory MM.

## Introduction

Multiple myeloma (MM) is a fatal plasma cell (PC) malignancy representing the second most common hematopoietic cancer. Unlike normal PCs, which are fully differentiated, antibody-producing B cells with a limited lifespan, malignant PCs retain their self-renewing capabilities and accumulate in the bone marrow resulting in malignancy [1], [2]. Over the last decade, remarkable advances have been made in the treatment of MM that have improved patient survival, including bone marrow transplant and the discovery of novel chemotherapeutic agents including proteasome inhibitors. Proteasome inhibitors block the ability of the proteasomal complex to degrade overabundant, misfolded or damaged polyubiquitinated proteins [3], [4]. The large-scale production of antibodies by PCs requires the systematic degradation of excess proteins to maintain cellular homeostasis making the proteasome complex a successful chemotherapeutic target for MM [5].

Bortezomib (Bz)/VELCADE® (Millennium Pharmaceuticals, Inc.) was the first clinically approved, specific inhibitor of the proteasome and is a member of a growing family of clinical proteasome inhibitors including next-generation compounds such as MLN9708/ixazomib (Millennium Pharmaceuticals, Inc.) and the recently FDA-approved carfilzomib (Onyx Pharmaceuticals) [5]. Bz reversibly inhibits the PSMB5 subunit of the proteasome, primarily targeting its chymotrypsin-like activity [6] and has been widely used to treat MM in combination with agents such as melphalan, dexamethasone, thalidomide and other newer IMiD-derivatives such as lenalidomide and pomalidomide [5].

MM patients treated with Bz alone or in combination with other agents have achieved high response rates [7]. Despite this initial success, the majority of patients eventually relapse; some maintaining sensitivity to further Bz-based therapy, while others develop refractory disease due to “acquired” drug resistance. Furthermore, approximately 20–30% of MM patients fail to initially respond to Bz [8] having primary refractory disease and, therefore, display “innate” resistance to the drug [9]. However, the similarities and differences between innate and acquired Bz resistance remain ill-defined. Moreover, there are no reliable diagnostic predictors to determine whether a patient will respond to Bz treatment. By the time MM patients are classified as drug resistant, their prognosis is often poor. Therefore, diagnostic tests that could predict Bz sensitivity or resistance prior to treatment as well as identification of novel therapies that could specifically target drug resistant cells are critically needed and could improve patient outcomes.

The goal of this study was to identify and validate those immunophenotypic markers that best distinguish Bz-sensitive from -resistant cells to establish signatures that predict Bz sensitivity. This would provide preclinical support for the development of a future diagnostic test for MM patients and to identify the potential for Bz re-sensitization. We utilized the previously described Bz-sensitive (BzS) and Bz-resistant (BzR) mouse cells lines [10] derived from tumors of the Bcl-X_L_/Myc double transgenic mouse model of PC malignancy [11]–[13]. We employ this model because PC tumor lines isolated from these mice closely resemble human MM based on gene expression profiling (GEP), chromosomal abnormalities and progression of disease in the bone marrow [10]–[13]. Here we identify the loss of certain PC maturation markers as a component of an immunophenotype that is associated with both innate and acquired Bz resistance. Treatment of Bz-resistant cells with lipopolysaccharide (LPS) re-established a differentiated PC immunophenotype and, most importantly, restored Bz sensitivity. This highly regulated PC maturation network may prove vulnerable in the case of Bz resistance and highlights that Bz resistance may not be permanently established but may be reversed by the proper combination of secondary therapy that force Bz-resistant cells toward PC maturation.

## Materials and Methods

### Mouse Tumor Cell Lines and Treatment Conditions

Mouse cell lines 595 BzS and 589 BzS were isolated from two independent Bcl-XL/Myc double transgenic mice. Bz resistant cell lines 595 BzR and 589 BzR were generated from these 595 BzS and 589 BzS lines as previously described [10]. Mouse cell lines were cultured in mouse PC media containing RPMI 1640 (Lonza, Allendale, NJ), 15% fetal bovine serum (FBS) (Cellgro, Mediatech, Manassas, VA), 25 mmol/L HEPES (Lonza), 1 mmol/L sodium pyruvate, 50 µmol/L beta-mercaptoethanol (Sigma-Aldrich, St. Louis, MO), 50 units/ml of penicillin and streptomycin (Thermo Fisher Scientific, Waltham, MA), 2 mmol/L _L_-glutamine (Gibco Life Technologies, Grand Island, NY) and 0.5 ng/ml interleukin (IL)-6 (R&D Systems, Minneapolis, MN). Cells were split every 3 days and maintained at concentrations between 2–5×10^6^ cells/mL. In some experiments, live cells were isolated by Ficoll-Hypaque (GE Healthcare, Piscataway, NJ) prior to analysis.

Bortezomib (Bz) (Millennium Pharmaceuticals, Inc., Cambridge, MA) was dissolved in serum-free RPMI 1640, and lipopolysaccharide (LPS) (E-Coli 0111:B4, Sigma-Aldrich) was dissolved in PBS. Bz and LPS were added to the media at the concentration and for the time indicated.

### Cytotoxicity Assay

Cells were cultured at 4×10^5^ cells/mL and treated with indicated concentrations of Bz. Cells were subjected to CellTiter-Glo® Luminescent cell viability assay according to manufacturer’s instructions (Promega, Madison, WI). Values were normalized to untreated controls. Where indicated, numbers of total live cells were determined by trypan blue exclusion and counting in triplicate using a hemocytometer.

### Fluorescence Analysis and Sorting

Cells were stained with the following anti-mouse antibodies: CD184/Cxcr4 PE (clone 2B11, also used for anti-human Cxcr4), CD69 FITC (clone H1.2F3), CD93 APC (clone AA4.1), CD20 PE (clone AISB12), CD22 FITC (clone 2D6), CD27 FITC (clone LG.7F9) (all from eBioscience, San Diego, CA), CD138/syndecan-1 PE (clone 281-2), CD19 PE (clone 1D3), B220 APC (clone RA3-6B2) (all from BD Biosciences, Franklin Lakes, NJ), IgM-FITC (SouthernBiotech, Birmingham, AL), CD38 (BioLegend, San Diego, CA) and analyzed using the FACSCalibur (BD Biosciences, Franklin Lakes, NJ). For fluorescence activated cell sorting experiments, at least 2×10^7^ cells were used, stained as described above using anti-mouse CD93 APC (clone AA4.1) and sorted using a FACSAria (BD Biosciences). All samples were normalized to an unstained or isotype stained control.

### Extraction of RNA and Quantitative RT-PCR

RNA was extracted using QIAshredder and RNeasy RNA purification columns (Qiagen, Valencia, CA). RNA was reverse transcribed using the Transcriptor First Strand cDNA Synthesis Kit (Roche, San Francisco, CA). Quantitative RT-PCR was performed in technical triplicate (*i*.*e*. on each cell line under the given condition) using the LightCycler 480 and Probes Master (Roche) using 45 cycles of 95°C for 10 sec and 55°C for 30 sec (single acquisition). The primers used are presented in Material and Methods S1. Data were analyzed using LightCycler 480 software (Roche), and relative fold changes were calculated using the 2^−ΔΔCt^ method with the *Gapd* reference gene for normalization.

The ratio of spliced to unspliced *Xbp-1* was determined by endpoint PCR using the GeneAmp PCR System 9700 (Applied Biosystems, Carlsbad, CA) and GoTaq Green DNA polymerase (Promega) using 35 cycles of 95°C for 30 sec, 50°C for 30 sec and 72°C for 1 min. The *Xbp-1* primers used were previously described [14] and have been included in Material and Methods S1. PCR products were resolved on a 2.5% agarose gel (BioExpress, Kaysville, UT), visualized using the ChemiDoc™ XRS+ Imager (Bio-Rad, Hercules, CA), and quantified by Image Lab Software.

### Statistical Methods

For all flow cytometry experiments, statistical significance where indicated was determined using a two-tailed Student’s t-test with biological triplicates as these were exploratory experiments. For all validation quantitative RT-PCR experiments, statistical significance where indicated was determined using a one-tailed Student’s t-test with experimental triplicates. In all cases, a p-value <0.05 was considered significant.

Gene expression profiles of CD138^+^ bone marrow PCs from MM patients enrolled in the APEX [15] phase 3 clinical trial were used for human validation in this study. PC purifications and gene expression profiling, using the Affymetrix U133A/B microarray (Affymetrix, Santa Clara, CA), were performed as previously described [15], [16]. Signal intensities were pre-processed and normalized by GCOS1.1 software (Affymetrix). Permutation analyses were performed to correlate *PRDM1* and *CD93* expression with patient survival in the APEX trial (n = 264). The presented p-value was based on a log-rank test of survival of the high and low expression groups (quartile 4 and quartile 1, respectively) in each dataset. All statistical analyses were performed with the use of the statistical software R (Version 2.6.2) (http://www.r-project.org). A p-value <0.05 was considered significant.

## Results

### Acquired Bortezomib Resistance is Associated with Immunophenotypic Changes

To identify immunophenotypic biomarkers in Bz-resistant PCs, we utilized previously described *in vitro* cell lines developed from the double transgenic Bcl-X_L_/Myc mouse model of PC malignancy [10]–[12]. In the study by Stessman, *et al.*, Bz-sensitive (BzS) mouse cell lines isolated from this model system were dose escalated with Bz *in vitro* to create Bz-resistant (BzR) daughter cell lines [10]. These BzS and BzR populations were further characterized using gene expression profiling revealing signatures of Bz sensitivity and resistance that were correlated to Bz response in a human clinical trial [10]. We selected the most Bz-resistant of these cell line pairs, cell line 589 (4.4-fold increase in IC_50_) and cell line 595 (4.9-fold increase in IC_50_) (Table 1), for further immunophenotypic characterization at the protein level of the cell-surface markers that were identified in the previous study [10].

**Table 1:** IC_50_ table for mouse cell lines *in vitro*.

Drug	595 BzS	595 BzR	589 BzS	589 BzR	589 I-BzR
Bortezomib, nM	23	122	22	96	82

**Figure 1:**
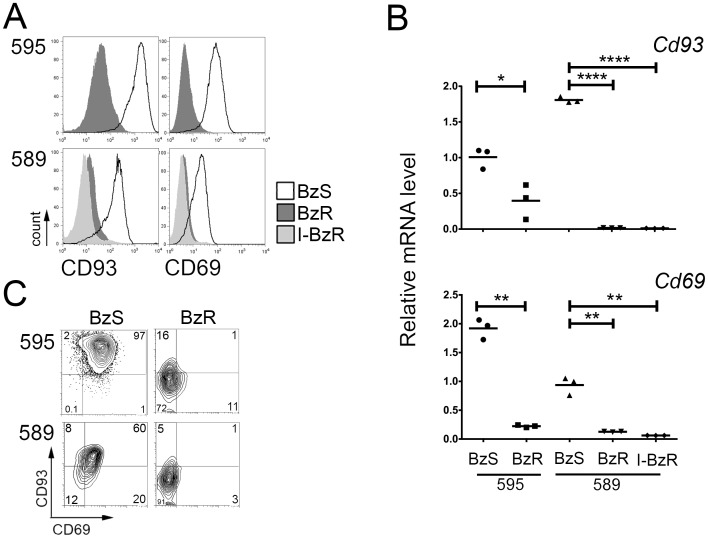
Immunophenotypic characterization of bortezomib resistant lines. A. Fluorescence-activated cell sorting analysis of BzS (solid black line), BzR (dark grey histogram) and I-BzR (light grey histogram) cells stained with indicated antibodies. B. Quantitative RT-PCR analysis of *Cd93* and *Cd69* relative mRNA expression in 595 BzS and BzR and 589 BzS, BzR, and I-BzR cell lines. Values were normalized to *Gapd* mRNA and error bars represent PCR triplicates. Significance was determined using a one-tailed Student’s t-test (*p<0.05; **p<0.01; ****p<0.0001). C. Fluorescence-activated cell sorting dot plot analysis of BzS and BzR cells double stained with CD93 and CD69 antibodies. Isotype controls for CD69 (FITC) and CD93 (APC) staining have been provided in Figure S1A.

The 589 and 595 cell line pairs were characterized by flow cytometry for a panel of 10 cell-surface proteins – CD93, CD69, CXCR4, CD20, CD19, CD22, CD38, CD138, B220, CD27– that were selected based on differential mRNA expression from the previous gene expression profiling study [10] and/or differential expression during normal B cell to PC maturation. Both the 589 and 595 BzS cell lines were found to be CD38^+^CD138^+^ (Figure S1A) and CD20^−^CD27^−^ (data not shown) compared to isotype controls (Figure S1A), characteristic of PCs [17]. Although the expression of most cell-surface markers remained unchanged between BzS and BzR cells (data not shown), the differences between these cell types were most well-defined by cell surface CD93 and CD69 protein expression (Figure 1A); therefore, we chose to focus on these two markers and their significance as biomarkers of Bz sensitivity.

These CD93 and CD69 cell-surface proteins have not been previously described in malignant human PCs. CD93, a known PC induction marker in mouse PC maturation, and CD69, an activation marker, had reduced expression in the BzR cell lines which was consistent with previously reported mRNA gene expression data [10]. CD93 and CD69 protein expression were reduced in BzR cell lines compared to the BzS matched controls (Figure 1A). Reduction in CD93 and CD69 cell surface protein expression also correlated with reduced expression of these genes at the mRNA level by quantitative RT-PCR (Figure 1B). Thus, using flow cytometry, BzS cells can be clearly and readily distinguished from BzR cells by CD93 and CD69 expression; BzS cells are CD93^+^CD69^+^ (double positive) and acquired BzR cells are CD93^−^CD69^−^ (double negative) (Figure 1C).

### Innate and Acquired Bortezomib Resistant Cells have Similar Immunophenotypes

Since MM cultures are known to be heterogeneous, we asked whether a side population of CD93^−^CD69^−^ double negative cells is present within drug naïve, BzS cultures that may display “innate” Bz resistance. While the majority of BzS cultures stained double positive for CD93 and CD69, approximately 0.1% of 595 and 12% of 589 BzS cells were double negative for CD93 and CD69 (Figure 1C, left panels). Characterization of 4 other independently-derived BzS cell lines from the same double transgenic mouse model also displayed 2–6% double negative cells (data not shown).

To further characterize this double negative drug naïve side population, we isolated CD93^−^CD69^−^ (I-BzR) cells from the 589 BzS culture, which displayed the highest percentage of double negative cells, by flow sorting. As anticipated, both CD69 and CD93 surface expression were reduced in the double negative population (Figure 1A). As in the Bz-selected BzR cell lines, *Cd69* and *Cd93* were also significantly reduced at the mRNA level in the drug naïve double negative cell line (Figure 1B). Next, we performed Bz dose response assays to test for Bz sensitivity in the CD93^−^CD69^−^ population. Dose response assays using Bz revealed that this drug naïve, double negative sorted population (I-BzR) had an IC_50_ (Table 1) and growth rate (Figure S1B) comparable to the acquired 589 BzR cell line which was selected with Bz. Interestingly, this apparent “innate” Bz-resistant immunophenotype was persistent as long as one year post-sort (data not shown) indicating that this population is stable and does not revert to CD93^+^CD69^+^ in the absence of stimulation, resembling a primary refractory disease phenotype. These results demonstrate that “innate” Bz-resistant cells (I-BzR) isolated from a drug naïve, heterogeneous culture display similar immunophenotypic characteristics to cells with an acquired (drug-selected) resistance to Bz and indicate that Bz-resistant cells are present in a heterogeneous population of drug naïve cells.

### Bortezomib Promotes Loss of CD93 and CD69

**Figure 2:**
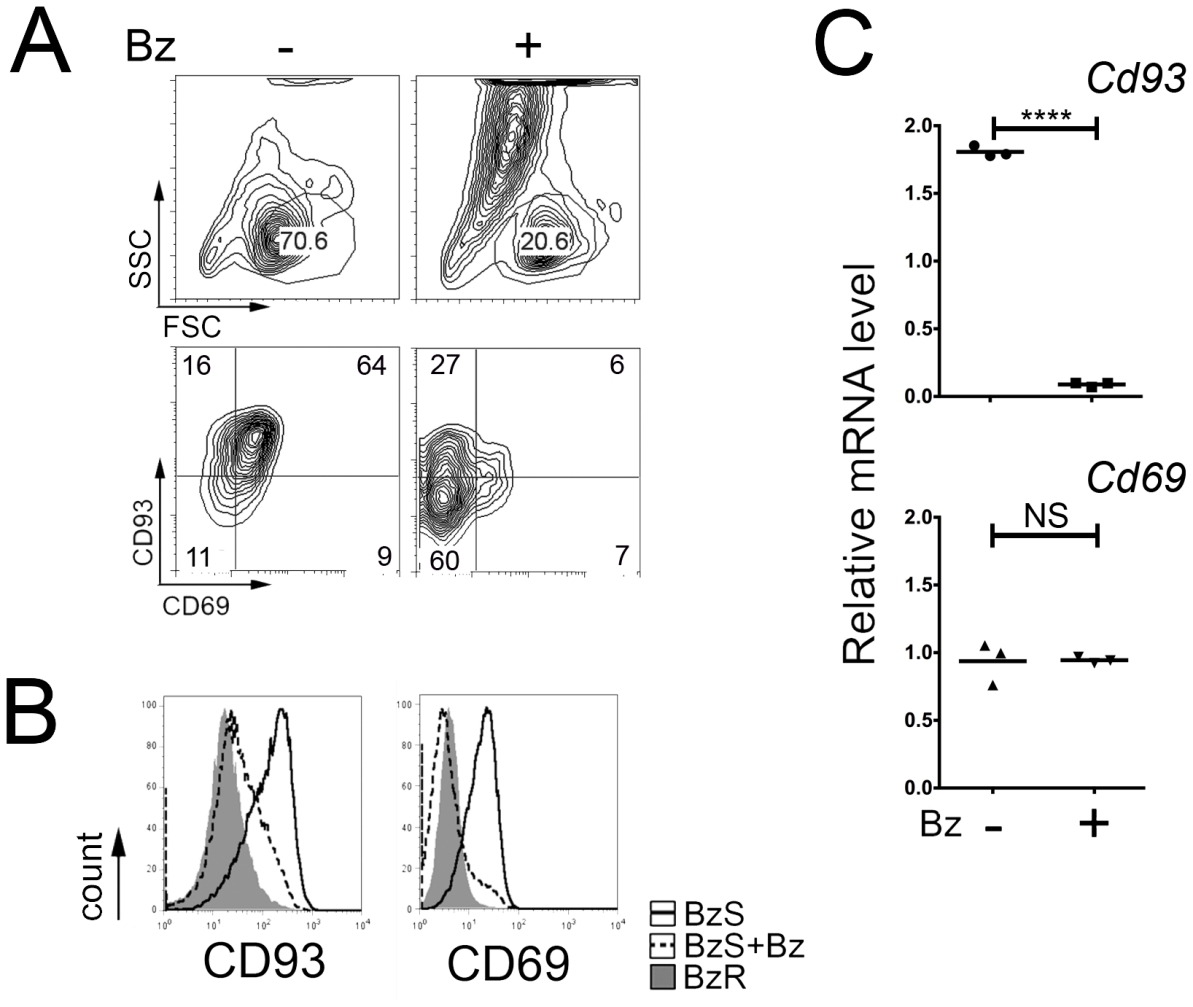
Bortezomib promotes loss of CD93 and CD69. A–B. Fluorescence-activated cell sorting analysis of 589 BzS cells in the presence (+) or absence (−) of 64 nM Bz for 48 hours. Live cells were gated based on FSC and SSC and dot plots representing CD93 and CD69 double stained populations in A. In B., histograms show live untreated BzS cells (solid black line), BzS cells treated with 64 nM Bz for 48 hours (dotted black line) and untreated BzR cells (dark grey histogram) stained with CD93 and CD69 antibodies. C. Quantitative RT-PCR analysis of *Cd93* and *Cd69* mRNA in 589 lines corresponding to Figure 2A–B. Values were normalized to *Gapd* mRNA and error bars represent PCR triplicates. Significance was determined using a one-tailed Student’s t-test (****p<0.0001; NS = not significant).

To characterize the emergence of the CD93^−^CD69^−^ double negative Bz-resistant population, CD93^+^CD69^+^589 BzS cells were treated with either a high or a low dose of Bz and the expression of CD93 and CD69 was evaluated. As expected, BzS cells treated with a high dose (64 nM) of Bz for 48 hours resulted in approximately 80% death (Figure 2A, Figure S1C). After gating on the live cells (Figure 2A, top panel), we observed decreased expression of CD93 and CD69 by flow cytometry compared to untreated controls (Figure 2A, bottom panels and Figure 2B) as the remaining live cells displayed a BzR cell immunophenotype. The retention of these double negative cells is likely not due to variable growth rates as CD93 and CD69 double positive and double negative populations divide at similar rates (0.8–0.9 cell divisions/day, Figure S1B). Quantitative RT-PCR analysis of BzS cells following shorter length, sub-lethal Bz treatment (33 nM for 24 hours), a condition not associated with cell death (Figure S1D), resulted in significantly reduced mRNA expression of CD93 while only a minor reduction of CD69 was observed (Figure 2C). These results demonstrate that Bz treatment promotes the emergence of the CD93 and CD69 double negative population within 48 hours and induces the loss of CD93 at the level of transcription within 24 hours of treatment.

### *CD93* Expression is Associated with *BLIMP-1* Expression in Human MM and Predicts Better Survival

**Figure 3:**
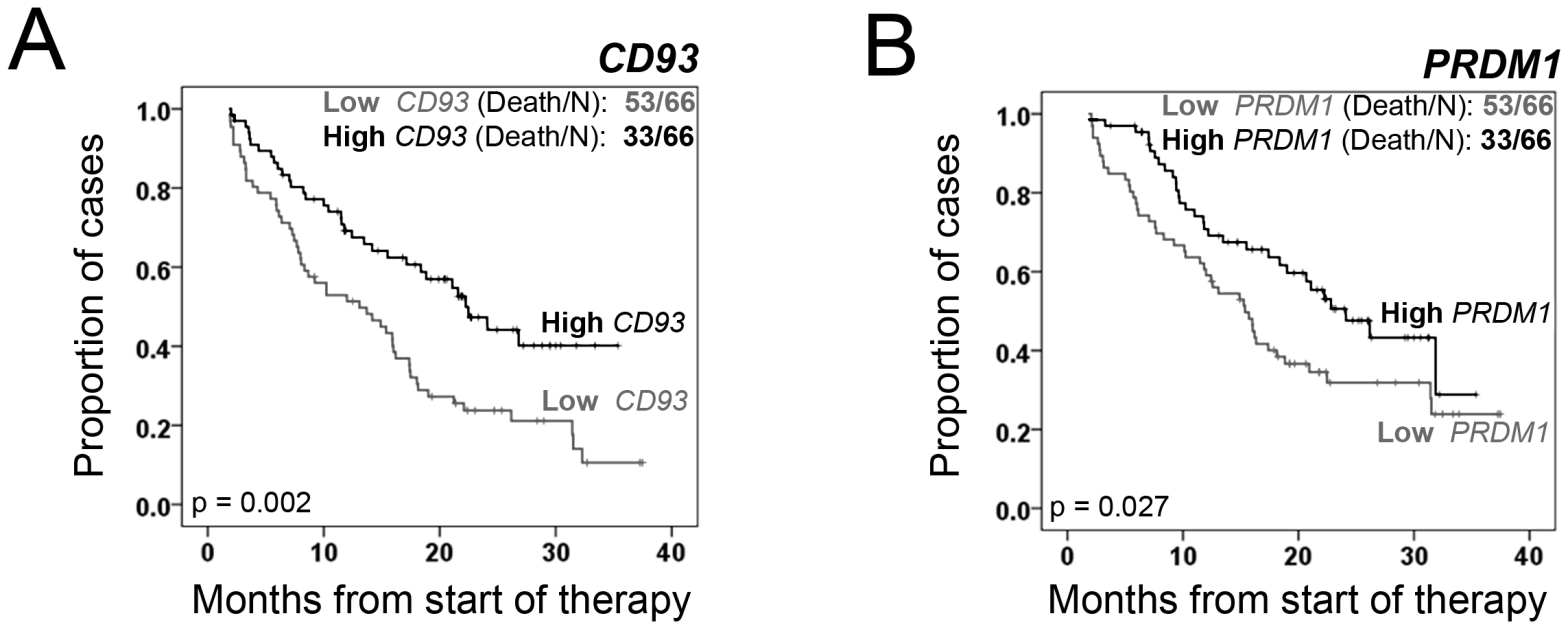
Low *CD93* and *BLIMP-1* expression are associated with poor clinical outcomes in patients treated with bortezomib. A–B. Survival analysis of high and low *CD93* (A) and *PRDM1* (*BLIMP-1)* (B) expressing MM patient groups taken from the APEX drug trial [15]. P-values represent significant differences by Log-rank test (p<0.05 was considered significant). The number of cases analyzed is indicated for each.

Since CD93 and CD69 were clear biomarkers of Bz sensitivity in the mouse system, we next sought to determine whether these markers are expressed in human MM and whether they are associated with differences in outcome in clinical trials of patients being treated with Bz. The APEX phase 3 clinical trial provides baseline GEP data from MM patients treated with either single-agent Bz or high-dose dexamethasone [15]. Using the mRNA expression values for *CD93*, we divided the patients into those with the highest and lowest *CD93* expression (*i.e.* quartile 4 and quartile 1, respectively). As a single biomarker, high *CD93* expression significantly distinguished those patients with better overall survival (p = 0.002) in the APEX trial data (Figure 3A). *PRDM1* (*BLIMP-1)*, a marker of mature PCs, as a single biomarker also significantly distinguished patients with better overall survival (p = 0.027) (Figure 3B). Consistent with previous studies in mouse PCs [18], high *CD93* expression was positively correlated with *BLIMP-1* expression in the MM patient samples (data not shown). Gene expression of *CD69* and other B cell maturation markers (including *PAX5*, *BCL6*, *XBP-1*, and *IRF4*) did not significantly predict differences in patient survival in the APEX trial. These data suggest that *CD93* may indeed be a marker of mature PCs in humans as it is in mice [18] and, furthermore, that a loss of the mature PC markers *CD93*, *BLIMP-1,* and the previously reported *CXCR4* [19], [20] may be predictive of poorer outcome in MM patients.

### Lipopolysaccharide Treatment Induces Expression of PC Maturation Markers and Re-sensitizes bortezomib-resistant cells to bortezomib

Reduction of CD93 (Figures 1 & 2) and the previously published CXCR4 [19] protein expression in the mouse BzR cells indicated that Bz resistance may be associated with a loss of PC maturation. Therefore, we hypothesized that by therapeutically targeting and promoting PC maturation of BzR cells that we could restore the immunophenotype of the BzS population and re-sensitize BzR cells to Bz-induced death. Further, the BzR phenotype in the 589 and 595 mouse cell lines is not associated with PSMB5 active site mutations [10] arguing that Bz resistance may be reversible.

**Figure 4:**
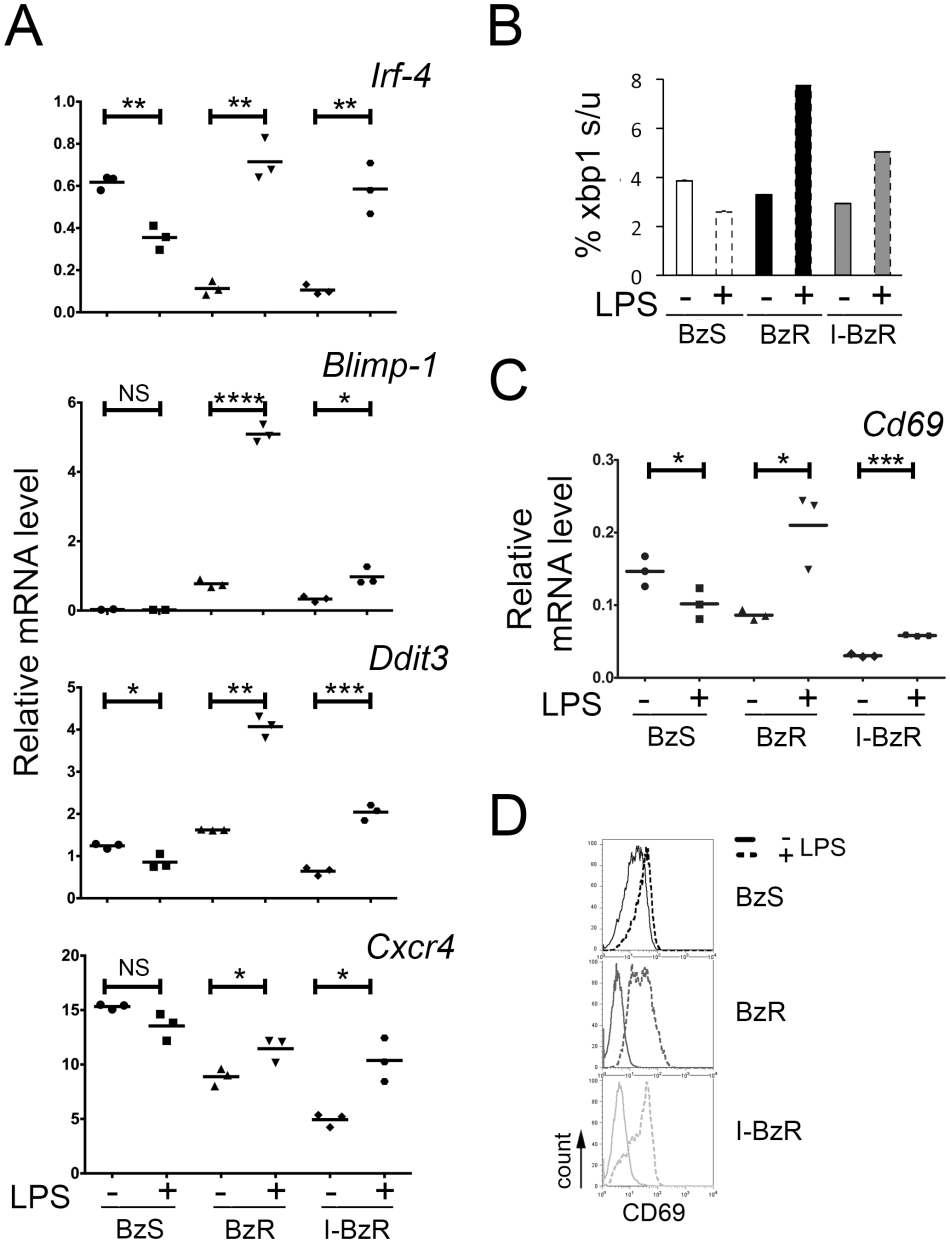
LPS induces plasma cell maturation in bortezomib-resistant cells. A. Quantitative RT-PCR analysis of *Irf-4*, *Blimp-1*, *Ddit3*, and *Cxcr4* mRNA expression in 589 untreated cells and following 72 hours of LPS treatment. Values were normalized to *Gapd* mRNA, and error bars represent PCR triplicates. Significance was determined using a one-tailed Student’s t-test (*p<0.05; **p<0.01; ***p<0.001; ****p<0.0001; NS = not significant). B. Densitometry values representing the percentage of spliced/unspliced (s/u) *Xbp-1* in 589 BzS, BzR and I-BzR cells untreated or treated with LPS for 72 hours. C. Quantitative RT-PCR analysis of *Cd69* mRNA expression in 589 untreated cells and 72 hour LPS-treated cells. Values were normalized to *Gapd* mRNA and error bars represent PCR triplicates. Significance was determined using a one-tailed Student’s t-test (*p<0.05; ***p<0.001). D. Fluorescence-activated cell sorting analysis of untreated (solid black line) BzS (top panel), BzR (middle panel), or I-BzR (bottom panel) and LPS-treated (dotted lines) cells stained for CD69 protein expression.

The toll like receptor (TLR)-4 ligand, bacterial lipopolysaccharide (LPS), is a known inducer of B cell maturation [21], [22]. Previous studies have shown that maturation of mature B cells to immunoglobulin-secreting PCs requires the coordinated induction of the transcription factor IRF-4 [23] and its downstream transcriptional target, BLIMP-1 [24] (Table 2). Consistent with these studies, LPS treatment of 589 cells for 72 hours resulted in a 6-fold increase in *Irf-4* in both derived BzR and I-BzR cells and a 3-fold (I-BzR) to 6-fold (BzR) increase in *Blimp-1* (Figure 4A) mRNA levels. Increased BLIMP-1 has also been positively correlated with the expression of DNA-damage-inducible transcript 3 (DDIT3) which is upregulated as part of the unfolded protein response during normal PC maturation [23] and CXCR4 which is expressed on mature PCs and aids in bone marrow homing [25] (Table 2). Consistent with this, we observed increased *Ddit3* and *Cxcr4* mRNA expression in BzR cells following LPS treatment (Figure 4A).

**Table 2:** Protein biomarkers with a described role in mouse plasma cell maturation.

Biomarker	Mature B cell	Plasma cell
BLIMP-1	–	+
IRF-4	–	+
DDIT3	+	++
XBP-1 (ratio s/u)^a^	–	+
Immunoglobulin	+	+++
CD93	–	+
CXCR4	–	+

a Refers to the ratio of spliced to unspliced *Xbp-1* transcript present within the cell.

BLIMP-1 is also required for XBP-1 induction and activation of the physiological unfolded protein response (UPR) pathway in preparation for immunoglobulin secretion [14], [24], [26]. This UPR pathway is responsible for further processing of *XBP-1* mRNA into its spliced form with the assistance of IRE1α [14] (Table 2). Consistent with LPS-induced PC maturation, we observed increased *Xbp-1* splicing in the BzR cells but not in the BzS population (Figure 4B). *Xbp-1* splicing corresponded to increased Ig kappa secretion only in the I-BzR population (Figure S2B). The lack of Ig kappa secretion (Figure S2B) and undetectable intracellular Ig kappa in the acquired BzR cells (data not shown) correlated with a deletion within the kappa gene locus as evidenced by array comparative genomic hybridization (data not shown). Interestingly, increased expression of *Irf-4*, *Blimp-1*, *Ddit3*, *Cxcr4* and spliced *Xbp-1*, all well-described indicators of PC maturation (Table 2), were only observed in BzR cells and did not increase in the LPS-treated BzS cells (Figure 4A–B) suggesting that the BzS population is insensitive to further increases in LPS-mediated PC maturation markers despite similar expression of *Tlr4* mRNA (data not shown).

We next asked whether LPS stimulation promoted the re-expression of the mouse Bz-resistant cell biomarkers CD93 and CD69 in BzR populations. We observed a significant increase in *Cd93* mRNA (Figure S2C) and cell surface expression (Figure S2D) only in the I-BzR population consistent with the modest increase in Ig kappa secretion (Figure S2B) validating the positive correlation between CD93 expression and Ig secretion, which has been previously reported [18]. LPS stimulation consistently resulted in significantly increased *Cd69* mRNA (Figure 4C) and cell-surface protein expression (Figure 4D) in both BzR cell lines suggesting that its expression is likely not linked to immunoglobulin secretion and further that CD69 may be a reliable marker of PC maturation in the mouse. In contrast, LPS stimulation modestly, but significantly, reduced CD69 mRNA in BzS cells despite a lack of reduction of CD69 protein on the cell surface suggesting further differential response to LPS stimulation in BzS versus BzR cell lines. The LPS-stimulated CD93^+^CD69^+^ immunophenotype in BzR cells was not maintained in the absence of LPS as these cultures drifted back to CD93^−^CD69^−^ and Bz resistance over the course of 21 drug-free days following the initial three-day LPS treatment (data not shown).

**Figure 5:**
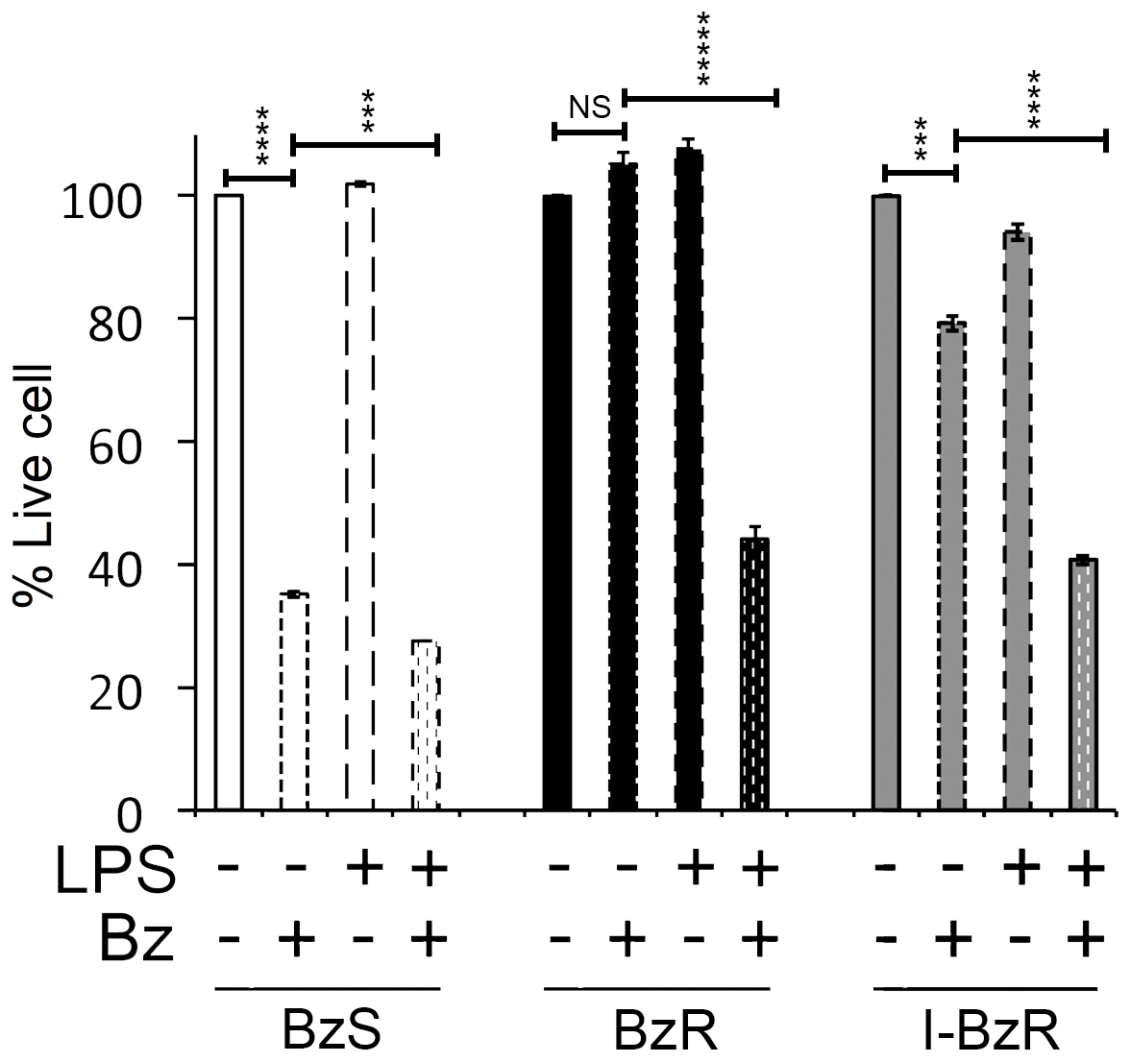
LPS re-sensitizes Bz-resistant cells to bortezomib treatment. 72 hour LPS-treated or untreated BzS, BzR, or I-BzR lines were ficolled and re-plated in the presence or absence of 60 nM Bz for 48 hours. The percentage of live cells determined by CellTiter-Glo® values normalized to untreated controls (*i.e.* In the absence of Bz, LPS-treated were normalized to non-LPS-treated cells, and following LPS treatment, Bz-treated were normalized to non-Bz-treated cells) are shown. Error bars represent three independent CellTiter-Glo® readings. Significance was determined using a one-tailed Student’s t-test (***p<0.001; ****p<1×10^−4^; *****p<1×10^−5^).

Finally, we asked whether re-maturation of the PC immunophenotype restores Bz sensitivity in BzR cells. Following LPS pre-treatment, live cells were re-plated for Bz treatment. In the absence of LPS treatment, consistent with our previous findings, we observed reduced viability in the BzS population and little death in BzR cell populations following Bz treatment (Figure 5). However, LPS pre-treatment followed directly by Bz treatment significantly reduced the viability in all cultures particularly those cultures that we had initially isolated as Bz-resistant populations (BzR and I-BzR) (Figure 5). We also observed increase sensitivity to Bz treatment in the 595 BzR cells line following LPS pre-treatment (data not shown). Thus, LPS pre-treatment promotes PC maturation and re-sensitizes BzR cells to bortezomib.

## Discussion

In this study we utilized tumor lines derived from the Bcl-X_L_/Myc transgenic mouse model of PC malignancy to immunophenotypically characterize neoplastic Bz-sensitive and -resistant PCs in order to identify biomarkers associated with acquired and innate Bz resistance. Although these pairs of cell lines shared many common markers of PC stage, we found that Bz-sensitive cells are predominately CD93^+^CD69^+^ (88–99.9%) (6 independently derived transgenic mouse lines analyzed), whereas BzR cells display a striking reduction in the expression of both of these markers. In fact, CD93 and CD69 are the two cell-surface proteins that best distinguished Bz-sensitive from -resistant cells (*e.g.* both innate and acquired).

Our analysis of an MM patient clinical trial (APEX) also revealed *CD93* as a biomarker of patient outcome. CD93 (C1qR_p_ or AA4.1) is a C-type lectin-like domain containing glycoprotein expressed on a variety of cell types and is known to play a role in phagocytosis and adhesion [27]. In the mouse, CD93 is expressed during early B cell maturation followed by downregulation on mature B cells and then re-expression on antibody-producing PC where it is required for the maintenance of long-lived PCs in the bone marrow [18]. Based on the data presented here, *CD93* may serve as a similar biomarker of a subset of PCs such as long-lived PCs in humans as well as a biomarker of better outcome in MM patients. We have also shown that low *CD93*, *BLIMP-1,* and previously that low *CXCR4* expression [19] are associated with poorer survival in MM patients treated with Bz all of which point toward a loss of some components of PC maturation as a mechanism of Bz resistance. Furthermore, analysis of the mouse cell lines has revealed a modest reduction in spliced *Xbp-1* in the Ig-secreting mouse 595 BzR cell line relative to its BzS counterpart but not in the 589 line which lacks heavy chain expression (Figure S2A), a requirement for maintaining *Xbp-1* expression in mature PCs [28]. A lack of spliced *XBP-1* has also been observed in PCs isolated from human Bz-resistant MM patients [29]. The common loss of select PC markers in both the human and mouse systems suggests that there is an association between Bz-resistance and a loss of PC maturation in Bz-resistant cells even though we do not observe a complete shift by GEP to an earlier stage of B cell maturation (data not shown). In the case of CD93 and CD69, the expression of these markers may be both selected for and modulated by Bz treatment. It is possible that additional cell-surface proteins that are required for PC maintenance that are downregulated in BzR cells may also be repressed by Bz-induced cellular reprogramming, a mechanism which has been recently suggested by others [30].

LPS stimulation induced the re-expression of CD93 in about 27% of I-BzR cells which was positively correlated with a 30% increase in Ig secretion, suggesting that these two events are likely occurring together within the CD93^+^ population. Since LPS pre-treatment did not uniformly increase CD93 expression, re-sensitization of BzR cells may not always correlate with CD93 expression. However, expression of the activation marker CD69 increased uniformly following LPS treatment but only in the BzR cell lines suggesting that CD69 may be the better marker for predicting Bz resistance although little is known about the function of this protein during PC maturation [31]. Future studies are required to determine the precise role of CD69 in Bz sensitivity, specifically, whether CD69 is simply an incidental biomarker or is playing a larger mechanistic role in resistance.

The re-sensitization of previously Bz-resistant cells to Bz following LPS pretreatment supports the hypothesis that BzR cells have lost some of their PC maturity distinct from Bz-sensitive cells. While LPS promoted PC maturation of BzR cells, BzS cells were unaffected by LPS stimulation similar to *in vitro* adapted plasmacytoma tumors [32]. Re-sensitization of Bz-resistant MM lines following PC maturation using 2-methoxyestrodiol or all-trans-retinoic acid has also been described [33]. Combined, these studies argue that augmentation of PC maturation may be a logical chemotherapeutic approach in Bz-refractory MM. Moreover, these results would suggest that emerging resistance may not always be the result of irreversible mutations, but an adaptive tumor response that can be reversed.

It is possible and, based on our data, likely that the loss of select PC maturation markers may serve as not only biomarkers of Bz resistance but as direct therapeutic targets in refractory MM. The loss of PC maturation may confer a selective advantage for evading Bz-mediated death via a reduction of the unfolded protein response (UPR). B cells, which express and secrete fewer immunoglobulin molecules than PCs, are less sensitive to endoplasmic reticulum stress and display a reduced UPR [14], [34]. We demonstrate that BzR cells secrete reduced immunoglobulin proteins compared to BzS cells, consistent with the observation that BzR cells have lost some of their PC maturity. This raises the possibility that BzR cells are resistant because of reduced antibody production suggested previously by others [35]. While LPS stimulation re-sensitized both BzR and I-BzR cells to Bz, re-sensitization correlated minimally with increased Ig secretion in I-BzR cells arguing, at least in this context, that Bz sensitivity does not always require Ig synthesis and secretion. On the other hand, LPS stimulation increased *Xbp-1* splicing and *DDIT3* expression, two components necessary for the UPR, suggesting that Bz sensitivity may require the initiation of the UPR signaling cascade which may occur independently of Ig synthesis.

These studies outline the immunophenotypic characterization of Bz-sensitive and -resistant PCs using a mouse model which has identified the loss of PC maturation in Bz-resistant cells compared to Bz-sensitive cells. By therapeutically forcing Bz-resistant cells to differentiate into PCs, we have shown that we can re-sensitize these cells to Bz treatment. Further exploration of this loss of PC maturation markers in Bz-resistant myeloma will require additional patient validation studies; however, this study may provide useful initial biomarkers for developing a diagnostic test to identify Bz resistance. The positive impact of such a diagnostic tool in patient care, if validated, could mean the early detection of Bz resistance and improved overall survival through individualized medical treatment. In addition, if in fact these immunophenotypic biomarkers are involved in the drug-resistant mechanism, this may provide novel drug targets for the synthesis of new compounds aimed at reversing resistance by promoting PC maturation prior to Bz treatment. This highlights a unique therapeutic option for the treatment of relapsed and primary refractory MM patients.

## Supporting Information

Figure S1 A. Fluorescence-activated cell sorting analysis of 595 BzS (solid black line) and 589 BzS (dotted black line) cells compared to GC B cell, CH12 (dark grey histogram) and plasmacytoma cell, MPC11 (light grey histogram), reference lines stained with indicated antibodies. Isotype controls shown for all fluorescence-activated cell sorting experiments. B. Total live 589 BzS, BzR, and I-BzR cells were determined by trypan blue exclusion by three independent cell counts. In C., 589 BzS cells were incubated in the presence or absence of 64 nM (high dose) Bz for 48 hours and in D., 589 BzS cells were incubated in the presence or absence of 33 nM (low dose) Bz for 24 hours. The percentages of live cells was determined by CellTiter-Glo® values normalized to untreated controls. Error bars represent three independent CellTiter-Glo® readings.
(TIF)

Figure S2 A. End-point RT-PCR analysis of *Xbp1* mRNA. Spliced *Xbp1s* is represented by a 26 bp smaller spliced product compared to unspliced, Xbp1u. B. ELISA of Ig kappa light chain secreted into the media following 72 hour LPS treatment, cells were ficolled and incubated for an additional 24 hours. The error bars represent three independent ELISA readings, and values were normalized to total live cells determined by CellTiter-Glo®. C. Quantitative RT-PCR analysis of *Cd93* mRNA in 589 untreated cells and 72 hour LPS-treated cells. Values were normalized to *Gapd* mRNA and error bars represent PCR triplicates. Significance was determined using a one-tailed Student’s t-test (**p<0.01; ***p<0.001). D. Fluorescence-activated cell sorting analysis of untreated BzS (top panel), BzR (middle panel) and I-BzR (bottom panel) LPS-treated cells co-stained with CD93 and CD38.
(TIF)

Material and Methods S1 Supporting Material and Methods.
(DOC)
