# Supplementary figures and images for: Bortezomib Resistance Can Be Reversed by Induced Expression of Plasma Cell Maturation Markers in a Mouse In Vitro Model of Multiple Myeloma
Source: PLoS One. 2013 Oct 29;8(10):e77608. doi: 10.1371/journal.pone.0077608 (PMC3812176; doi:10.1371/journal.pone.0077608)

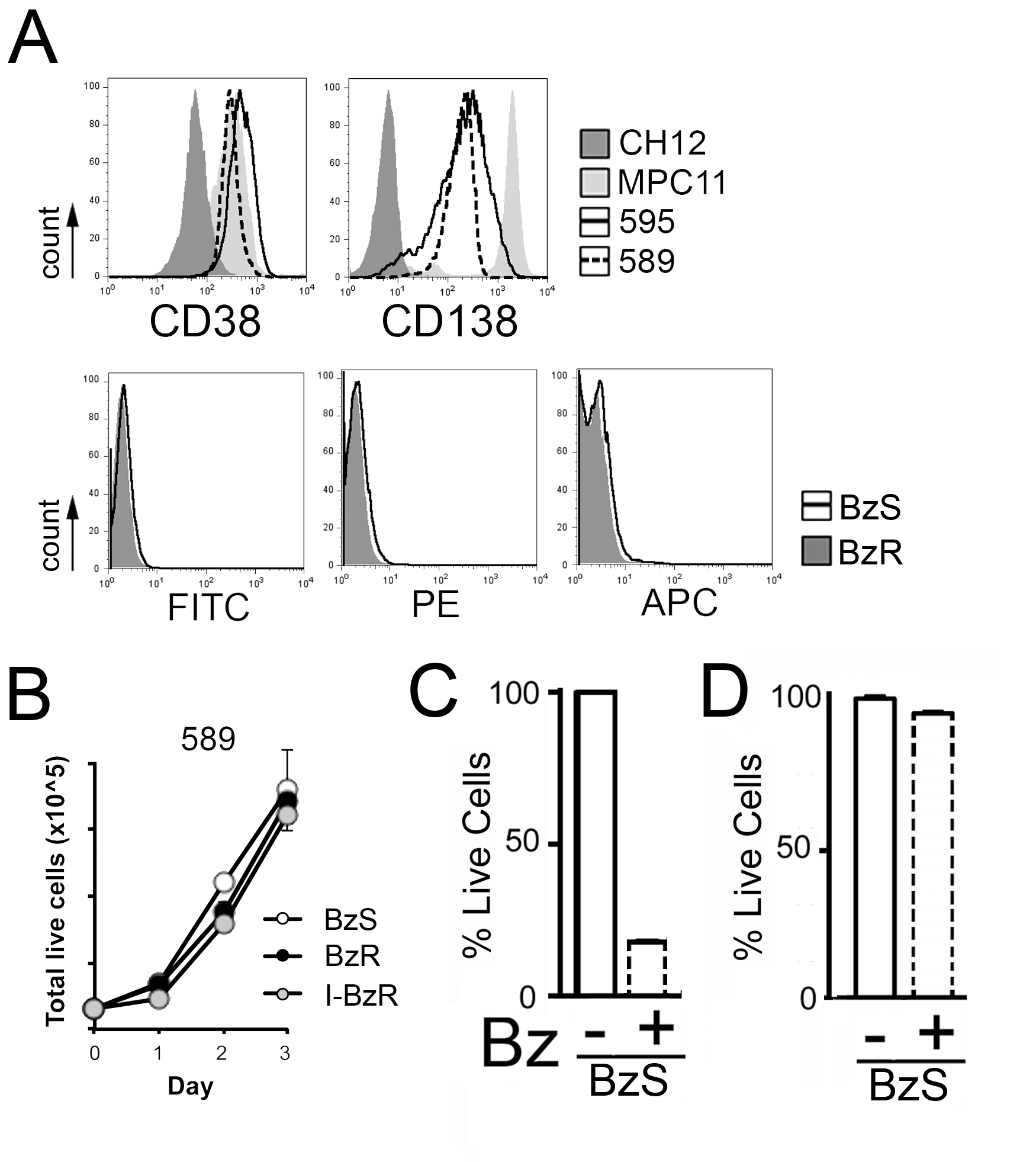

Supplement: Figure S1 — A. Fluorescence-activated cell sorting analysis of 595 BzS (solid black line) and 589 BzS (dotted black line) cells compared to GC B cell, CH12 (dark grey histogram) and plasmacytoma cell, MPC11 (light grey histogram), reference lines stained with indicated antibodies. Isotype controls shown for all fluorescence-activated cell sorting experiments. B. Total live 589 BzS, BzR, and I-BzR cells were determined by trypan blue exclusion by three independent cell counts. In C., 589 BzS cells were incubated in the presence or absence of 64 nM (high dose) Bz for 48 hours and in D., 589 BzS cells were incubated in the presence or absence of 33 nM (low dose) Bz for 24 hours. The percentages of live cells was determined by CellTiter-Glo® values normalized to untreated controls. Error bars represent three independent CellTiter-Glo® readings. (TIF) [file pone.0077608.s001.tif]

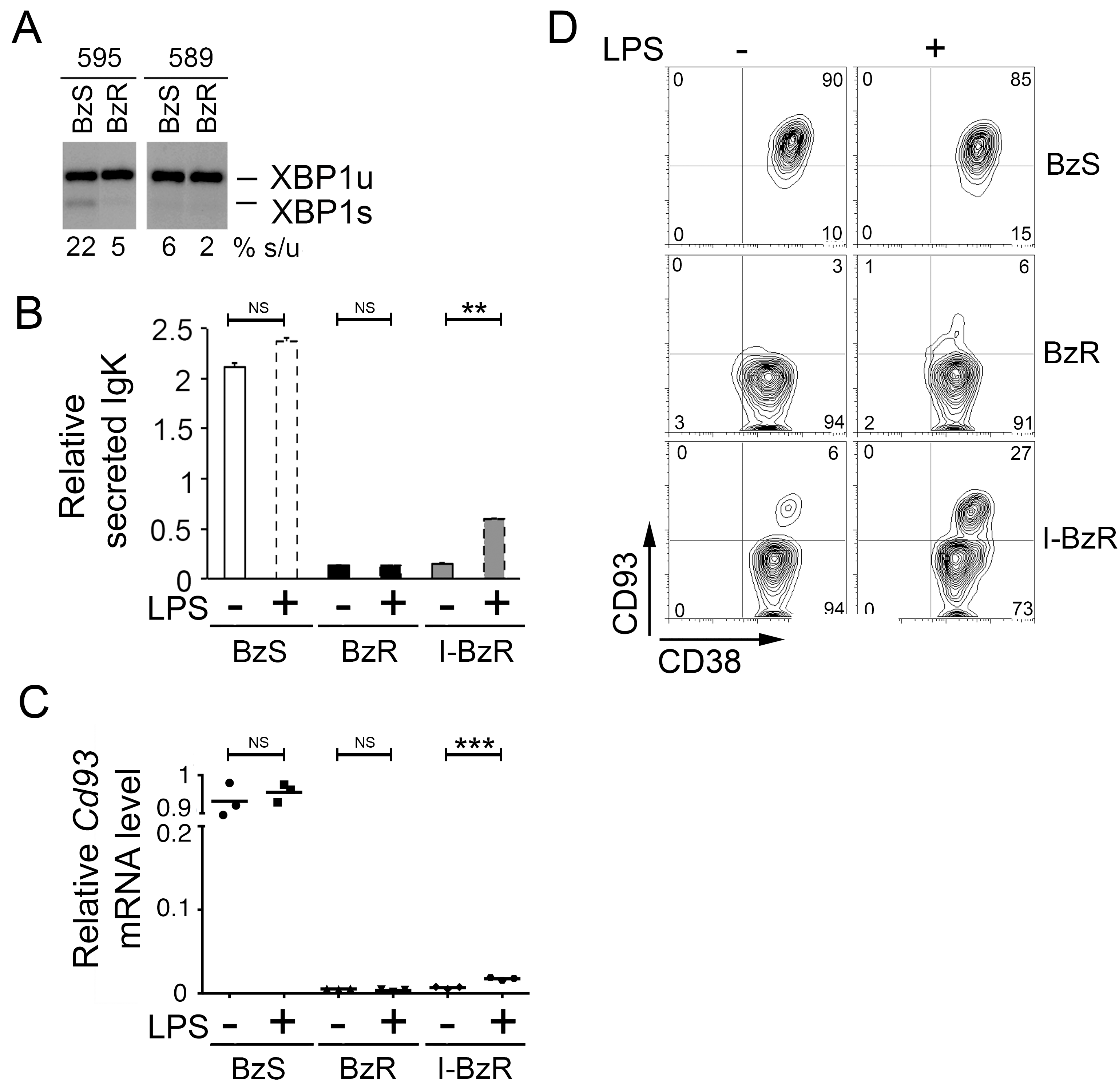

Supplement: Figure S2 — A. End-point RT-PCR analysis of Xbp1 mRNA. Spliced Xbp1s is represented by a 26 bp smaller spliced product compared to unspliced, Xbp1u. B. ELISA of Ig kappa light chain secreted into the media following 72 hour LPS treatment, cells were ficolled and incubated for an additional 24 hours. The error bars represent three independent ELISA readings, and values were normalized to total live cells determined by CellTiter-Glo®. C. Quantitative RT-PCR analysis of Cd93 mRNA in 589 untreated cells and 72 hour LPS-treated cells. Values were normalized to Gapd mRNA and error bars represent PCR triplicates. Significance was determined using a one-tailed Student’s t-test (**p<0.01; ***p<0.001). D. Fluorescence-activated cell sorting analysis of untreated BzS (top panel), BzR (middle panel) and I-BzR (bottom panel) LPS-treated cells co-stained with CD93 and CD38. (TIF) [file pone.0077608.s002.tif]
