# Supplementary material for: Bortezomib Resistance Can Be Reversed by Induced Expression of Plasma Cell Maturation Markers in a Mouse In Vitro Model of Multiple Myeloma
Source: PLoS One. 2013 Oct 29;8(10):e77608. doi: 10.1371/journal.pone.0077608 (PMC3812176; doi:10.1371/journal.pone.0077608)
Supplement: Material and Methods S1 — Supporting Material and Methods. (DOC) [file pone.0077608.s003.doc]

**SUPPORTING MATERIAL AND METHODS**

**Cell lines and culture.** CH12F3-2 (CH12) and MPC11 were kindly provided by Matthew Scharff (Albert Einstein College of Medicine, Bronx, NY). CH12 cells were maintained in RPMI 1640 supplemented with 10% FBS, 50 μmol/L 2-mercaptoethanol and 5% NCTC (BioWhittaker, Walkersville, MD), and MPC11 cells were cultured in murine PC media without IL-6.

**Quantitative RT-PCR primers.** The following primers were used: Cd93, 5’-TGAAATAGACGCCCTGAAAAC-3’ (5’ primer), 5’-AATCAAAGCCTGGGTTTAGGA-3’ (3’ primer); Cd69, 5’-GGAAAATAGCTCTTCACATCTGG-3’ (5’primer), 5’-TGATGCTTCTCAAAATGTATACTGG-3’ (3’ primer); Irf4, 5’-ACAGCACCTTATGGCTCTCTG-3’ (5’ primer), 5’-ATGGGGTGGCATCATGTAGT-3’ (3’ primer); Blimp-1, 5’-TGCGGAGAGGCTCCACTA-3’ (5’ primer), 5’-TGGGTTGCTTTCCGTTTG-3’ (3’ primer); Ddit3 (Chop), 5’-GCGACAGAGCCAGAATAACA-3’ (5’ primer), 5’-GATGCACTTCCTTCTGGAACA-3’ (3’ primer); Cxcr4, 5’- tggaaccgatcagtgtgagt-3' (5’ primer), 5’- gggcaggaagatcctattga-3’ (3’ primer). The primers included in the Universal ProbeLibrary Mouse Gapd Gene Assay (Roche) were used as the reference gene.

**Xbp1 primers.** Xbp1, 5’-ACACGCTTGGGAATGGACAC-3’ (5’ primer), 5’-CCATGGGAAGATGTTCTGGG-3’ (3’ primer).

**Enzyme linked immunosorbant assay (ELISA).** Cells (4 × 10^5^ cells/ml) were plated in mouse PC media for 48 hours. Supernatants were harvested and cells were lysed using buffer containing 10 mM Tris (pH 7.8), 150 mM NaCl, 1 mM EDTA, 1% Nonidet P-40. Ig kappa ELISA (Thermo Fisher Scientific) was performed according to the manufacturer’s instructions, and Ig kappa levels were determined by subtracting the value from medium alone or lysis buffer control and normalized according to CellTiter-Glo® luminescent values.
